# Focus on Apoptosis to Decipher How Alcohol and Many Other Drugs Disrupt Brain Development

**DOI:** 10.3389/fped.2014.00081

**Published:** 2014-08-04

**Authors:** John W. Olney

**Affiliations:** ^1^Department of Psychiatry, Washington University School of Medicine, St. Louis, MO, USA

**Keywords:** apoptosis, developing brain, neurons, oligodendrocytes, alcohol, sedatives, anesthetics, anti-epileptics

Maternal ingestion of alcohol during pregnancy can cause a disability syndrome termed fetal alcohol spectrum disorder (FASD), which may include craniofacial malformations, gross structural brain pathology, and a variety of long-term neuropsychiatric disturbances, or it may consist of subtle brain changes and neuropsychiatric disturbances in the relative absence of gross dysmorphogenic features. Based on a large body of recent evidence, we have proposed (1) that most, if not all, of the deleterious effects of alcohol on the developing brain can be explained by a single mechanism. Alcohol has apoptogenic properties that cause large numbers of CNS progenitor cells, or fully differentiated brain cells (depending on developmental age at time of alcohol exposure) to commit suicide and be deleted from the pool of cells that would ordinarily survive and contribute to the normal functions of the brain. If excessive cell suicide is triggered by alcohol in a very early stage of development, the result, as Sulik and colleagues have shown (2), will be gross dysmorphogenic anomalies (e.g., craniofacial and midline brain anomalies), because the cells deleted are progenitor cells that are responsible for generating cell populations that comprise the building blocks of these craniofacial and brain structures. But if, as we have demonstrated (3–5), alcohol triggers suicide of CNS cells in later stages of development after these cells are already differentiating into neurons and glia, the result will be a reduced number of brain cells, derangement of brain circuitry, and various neuropsychiatric disturbances, depending on which populations of cells have been deleted and what combination of synaptic connections have been disrupted or destroyed.

Alcohol’s apoptogenic action is linked to its NMDA glutamate antagonist and GABA_A_ agonist properties. Many other drugs that have one or both of these properties also trigger developmental apoptosis, including other drugs of abuse (phencyclidine, ketamine, benzodiazepines, and barbiturates), and many drugs used in obstetric and pediatric medicine [all sedative/anesthetic drugs (SADs), and most anti-epileptic drugs (AEDs)] (3, 6–8). It was demonstrated quantitatively in early studies that neurons are permanently deleted from the developing brain by exposure to these drugs, and that brain volume is permanently reduced and synaptic ultrastructure disrupted. No region of the central nervous system is totally spared, in that the degenerative response has been demonstrated in neurons distributed widely throughout the forebrain, midbrain, cerebellum, brainstem, spinal cord, and retina (3, 4, 9–11). Although alcohol’s apoptogenic action was originally thought to impinge only on neurons, it is now well established that oligodendrocytes (oligos), distributed diffusely throughout the white matter, also succumb to apoptosis following developmental exposure to alcohol or to SADs or AEDs (12–16). The injury induced by apoptogenic drugs is dose and developmental age-dependent, with several different patterns of neuronal degeneration observed, depending on developmental age at time of drug exposure. The cell death process involves Bax-mediated extra-mitochondrial leakage of cytochrome *c* (17), which is followed by a sequence of changes culminating in the activation of caspase-3 (5, 18). An important property that apoptogenic drugs have in common is that they rapidly suppress phosphorylation of extracellular signal-regulated kinases (ERK) (signaling system that regulates cell survival) in the *in vivo* developing brain. This has potentially important implications for preventing this type of brain injury, in that lithium counteracts the suppressant action of apoptogenic drugs on pERK (19), and also protects against apoptogenic injury induced by these drugs in the infant mouse (19–21) or infant monkey brain (22).

The developing rhesus macaque brain is quite sensitive to the toxic action of apoptogenic drugs, and in both rodents and monkeys two specific cell types are affected – neurons and oligos – and the mode of cell death for both cell types is apoptosis. Many of the structural brain changes reported in children with FASD are also seen in the brains of rodents and monkeys following exposure to alcohol and related apoptogenic drugs [illustrated extensively in Ref. (1)]. A prime example of a prominent structural brain change caused by alcohol and all other apoptogenic drugs following exposure of the primate brain in the early third trimester is loss of basal ganglia (BG) neuronal mass. This has long been recognized as a prominent finding in children who were exposed *in utero* to alcohol (23, 24), and also has been reported in children who were exposed to AEDs in the third trimester of gestation (25), and in premature infants who have learning disability following exposure to surgical anesthesia (26) or following prolonged sedation in the neonatal intensive care unit (27).

The window of vulnerability in primates appears to be very similar for all of these drugs. Valproate, an AED with very strong apoptogenic properties (7), mimics alcohol in causing craniofacial and midline brain anomalies following human exposure in the first trimester (28), and in causing a large IQ deficit following human exposure in the third trimester (29, 30). SADs have not been studied adequately for early dysmorphic effects, but we have shown that alcohol and numerous SADs (isoflurane, propofol, ketamine, benzodiazepines, and barbiturates) trigger a robust apoptosis response in the fetal monkey brain on gestational days 100–120 (comparable to human late second trimester), and vulnerability continues throughout the third trimester and up to a yet to be established age after birth (12–16, 31, 32). Mounting evidence from animal studies prompted a series of recent human studies, which have documented that brief anesthesia exposure of premature infants (26), or full term human infants (33–40) is associated with increased risk for neurocognitive deficits. Thus, it is clear that apoptogenicity is a property that alcohol and certain other drugs have in common, and emerging evidence suggests that in both early and late gestation these drugs have the potential to cause FASD-like structural brain changes and FASD-like neurodevelopmental disability syndromes. Available evidence suggests that FASD syndromes induced by anesthetic drugs are usually less severe than the syndrome that alcohol often causes, the obvious reason being that pregnant mothers who have a strong alcohol habit expose their fetuses multiple times during gestation to prolonged “binge” blood levels of alcohol, whereas the vast majority of human infants or fetuses who are exposed to anesthetic drugs are exposed only once for a relatively brief duration. Consistent with this thesis, the numerous human studies cited above are in good agreement that risk for poor neurocognitive outcome is greater following multiple anesthesia exposures than following a single exposure.

Although many mechanisms have been proposed to explain the FASD syndrome, the only mechanism identified, thus far, that can actually explain most if not all of the brain and behavioral pathology comprising that syndrome can be summed up in a single word – apoptosis. Within only a few hours after alcohol enters the developing brain, millions of brain cells that were on a healthy survival track, suddenly become derailed and commit suicide. The cells that die belong to both the neuronal and oligo lineages. Oligos are vitally important for normal neuronal function. Although widespread loss of neurons, or their progenitors, from the developing brain would be a sufficient mechanism to explain the signs and symptoms of FASD, simultaneous deletion of oligos, or their progenitors, makes the case even stronger for apoptosis as a single primary mechanism that can explain all features of the FASD syndrome. Once the apoptotic deletion of neurons and oligos (or their precursors) has occurred as the primary injurious event, there are numerous secondary mechanisms that come into play as the brain attempts to compensate for the disruptive influence of this primary injury. For example, loss of neurons causes an impoverishment of dendritic fields for receiving synaptic inputs from incoming axons, and loss of neurons also means there will be fewer axons to establish those synaptic contacts (41, 42). Developing brain networks must reconstitute and reorganize themselves to cope with this primary insult. Researchers can spend lifetimes studying the myriad steps in this reorganization process, but identifying these many features of the deranged and reorganized circuitry, will not yield insights necessary for preventing alcohol (or SADs and AEDs) from causing the initial injury and consequent derangements. The scenario I have just described pertains to a single episode of alcohol exposure. Consider how complicated the reorganization task will be for the brain of a fetus whose mother heavily abuses alcohol multiple times, both early and late, during pregnancy. Again, much time can be spent in studying this multi-layered complex reorganization process, but if the end goal is to learn how to prevent this type of developmental injury, the time will be better spent focusing on apoptosis as the primary cause, and deciphering the molecular mechanisms by which alcohol (or SADs and AEDs) unleash the apoptosis cascade. A better understanding of these mechanisms can lead to effective methods for preventing apoptogenic drugs from injuring the developing brain.

## Conflict of Interest Statement

The author declares that the research was conducted in the absence of any commercial or financial relationships that could be construed as a potential conflict of interest.
